# Narrow-linewidth monolithic topological interface state extended laser with optical injection locking

**DOI:** 10.1126/sciadv.ady8963

**Published:** 2025-09-10

**Authors:** Xiao Sun, Zhibo Li, Yiming Sun, John H. Marsh, David R.S. Cumming, Stephen J. Sweeney, Anthony E. Kelly, Lianping Hou

**Affiliations:** James Watt School of Engineering, University of Glasgow, Glasgow G12 8QQ, UK.

## Abstract

Narrow-linewidth lasers are essential for coherent optical applications, including communications, metrology, and sensing. Although compact semiconductor lasers with narrow linewidths have been demonstrated, achieving high spectral purity generally necessitates passive external cavities based on photonic integrated circuits. This study presents a theoretical and experimental demonstration of a monolithic optical injection locking topological interface state extended (MOIL-TISE) laser. By monolithically integrating a TISE laser with a micro-ring resonator on an AlGaInAs multiple quantum-well platform, the proposed device achieves efficient photon injection and linewidth narrowing. Experimental characterization indicates stable single-mode operation over a wide injection current range (65 to 300 milliamperes), exhibiting a side-mode suppression ratio exceeding 50 decibels. The laser’s Voigt linewidth was reduced from 2 megahertz to 4.2 kilohertz, with an intrinsic linewidth of 983 hertz extracted from power spectrum density, underscoring the MOIL-TISE laser’s promise for coherent communications and modulation-free quantum key distribution applications.

## Teaser

A narrow-linewidth laser is monolithically integrated with both a topological state extended cavity and a micro-ring resonator.

## INTRODUCTION

Lasers with high spectral purity are critical for numerous applications, including sensing and spectroscopy (*1*, *2*), optical clocks (*3*, *4*), light detection and ranging (*5*, *6*), and microwave photonic devices (*7*–*9*). Moreover, the demand for low-frequency noise is paramount in high data rate optical communications, particularly in coherent systems (*10*–*14*). Although conventional all-solid-state (*15*) and fiber lasers (*16*) offer excellent performance, their reliance on discrete, bulky components and low-energy conversion efficiency hampers on-chip integration. The π-phase–shifted distributed feedback (DFB) semiconductor lasers, by contrast, are more amenable to integration but inherently exhibit linewidths in the megahertz range (*17*), primarily due to their short cavity lengths and limited cavity quality factors. This makes them unsuitable for high-speed modulation formats such as 16-quadrature amplitude modulation (16-QAM) or 64-QAM, which require laser linewidths below 100 kHz.

To address these limitations, prior research has demonstrated that coupling a DFB semiconductor laser with a high-Q external optical cavity can create a self-injection–locked (SIL) system. The resulting narrow-band filtering feedback from the external cavity substantially suppresses the laser’s phase and frequency noise, thus enhancing its frequency stability. Previous studies have explored various external cavity configurations, such as Fabry-Perot (F-P) etalons (*18*, *19*), fiber ring resonators (*20*), and whispering gallery mode resonators (WGMRs) (*17*). However, many of these implementations still rely on optical fibers to connect the external cavities, constraining the potential for full on-chip integration.

For on-chip integration, a common strategy involves the hybrid integration of a DFB laser with a micro-ring resonator (MRR) on silicon on insulator (SOI) (*21*–*23*), lithium niobate on insulator (LNOI) (*24*–*26*), and Si_3_N_4_-based platforms. Si_3_N_4_ (*27*–*31*) is widely used for external optical cavities due to its low loss. However, achieving low thermal fluctuation noise typically requires a relatively large footprint (*27*, *28*), which can limit integration density. Moreover, the phase tuning of Si_3_N_4_ is mainly based on the thermo-optic effect, which limits the tuning speed to tens of kilohertz (*32*). Rayleigh backscattering in the high-*Q* (∼10^7^) MRR can reduce the laser linewidth to the subkilohertz level. However, under weak feedback conditions (typically <−20 dB), SIL is highly sensitive to feedback phase and external perturbations. Moreover, the intensity of Rayleigh backscattered light reinjected into the DFB laser is limited by the coupling efficiency between the DFB device and the external cavity. This coupling is practically limited by the challenges of heterogeneously integrating III to V materials onto silicon platforms. Specifically, realizing effective SIL and external cavity laser (ECL) designs require high efficiency coupling components, such as spot size converters (SSCs). Moreover, heterogeneous integration complicates the testing of individual components before their integration into complex systems. This necessitates stringent process control to ensure high yield while also increasing fabrication complexity and cost.

Topological photonic crystals (T-PhCs) have emerged as a revolutionary concept in the field of photonics, offering unique advantages for the manipulation and control of light. The design of T-PhC is based on exciting topological edge states at the interface between photonic structures with distinct topological Zak phases. This topological interface state (TIS) exhibits a single longitudinal mode (*33*–*35*), making it ideal for single-mode laser applications. In our previous work, we theoretically and experimentally demonstrated the TIS extended (TISE) laser structure (*36*), which reduced the intrinsic linewidth of the laser without requiring an external cavity. Experimental characterization confirmed that the TISE cavity effectively suppresses nonuniform carrier depletion and spatial-hole-burning–induced gain defects at high injection currents, thereby substantially enhancing robustness to material variations. The optimized TISE cavity design offered three key advantages: a reduced optical linewidth, suppression of mode hopping, and effective side-mode apodization. Furthermore, the enlarged modal footprint and robust optical confinement of the TISE cavity provided scalability and enabled efficient evanescent coupling with other integrated photonic components. However, because of the limited quality factor of the laser cavity, the intrinsic linewidth was only reduced to 150 kHz—still above the 100 kHz required for high-speed modulation in coherent optical communication. Thus, the further optimization of the TISE cavity is needed to achieve a narrower linewidth.

Here, we explore integrating TISE concepts into monolithic optical injection locking (MOIL) laser designs. We propose and experimentally demonstrate a MOIL TISE (MOIL-TISE) laser by monolithically integrating a TISE laser and an MRR on an AlGaInAs/InP multiquantum well (MQW) platform. The TISE laser establishes a central topological interface extended state, resulting in uniform photon density distribution and enhanced laser-MRR optical evanescent coupling. We fabricated a sidewall MOIL-TISE laser achieving single-mode operation with a DFB current range from threshold 65 to 300 mA and an side-mode suppression ratio (SMSR) of >50 dB without antireflective (AR) coatings. Under injection locking, the Voigt-fitted linewidth narrows substantially from 2 MHz to 4.2 kHz. The intrinsic linewidth, measured from the frequency noise power spectral density (FN-PSD), is as narrow as 983 Hz. The MOIL-TISE laser has a compact footprint of 1000 μm by 0.4 μm, which is substantially smaller than equivalent hybrid Si_3_N_4_ or SOI SIL/ECL lasers. We investigated its phase characteristic through coherent one-way (COW) protocol modulation and a phase-switching modulation proof-of-concept experiment, confirming transitions between random-phase and phase-locked states. The MOIL-TISE laser system offers excellent single-mode and narrow-linewidth performance within a compact and simplified fabrication process, requiring only one metalorganic vapor-phase epitaxy (MOVPE) growth and a single inductively coupled plasma (ICP) etching step. This highlights its strong potential for integrated photonic platforms, coherent optical systems, and modulation-free quantum key distribution (QKD) encoding.

## RESULTS

**Fig. 1.**
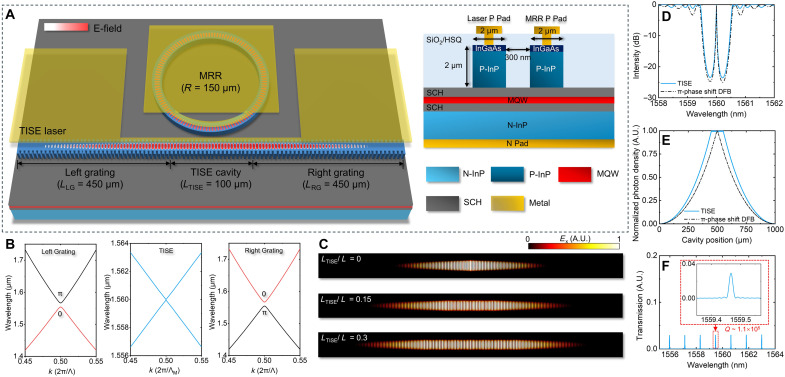
MOIL-TISE laser. (**A**) Structure and electrical field distribution of the MOIL-TISE laser. (**B**) Calculated band diagram band structures of the left grating, TISE grating, and right grating. (**C**) Normalized electric field distributions for *L*_TISE_/*L* = 0, 0.15, and 0.3. (**D**) Transmission spectrum and (**E**) normalized photon distribution in the TISE laser compared to the conventional π-phase shift DFB laser. (**F**) Transmission spectrum from the TISE laser to MRR.

Figure 1A illustrates the structure of the MOIL-TISE laser, which integrates a TISE laser with an MRR on an AlGaInAs MQW platform whose gain spectrum is centered ~1560 nm. The grating ridge waveguide was designed with a width of 2.0 μm and a ridge height of 2.0 μm. The TISE laser, measuring 1000 μm in total length (*L* = *L*_LG_ + *L*_RG_ + *L*_TISE_), comprises symmetrically inverted left and right reflective grating mirrors (*L*_LG_ = *L*_RG_ = 450 μm) flanking a central TISE cavity (*L*_TISE_ = 100 μm). The TISE cavity uses a third-order grating with a period of 720 nm. In this design, we use a uniform grating modulation approach, as previously described in (*36*), with a modulation period length of 3.6 μm. The cavity comprises 28 modulation periods in total. Compared to sampled grating modulation, the uniform grating modulation provides improved photon density uniformity (see the “Comparison of uniform grating and sampling grating modulations” section in the Supplementary Materials). The band-degenerate TISE cavity provides topological protection of the phase transition between the left and right gratings as shown in Fig. 1B. The Zak phase is quantized at either 0 or π when the origin is chosen to be one of the inversion centers, and the photonic bandgap near the center wavelength emerges and exhibits the nontrivial distinct topological index, Zak phase, associated with the left and right gratings. As a result, a domain-wall interface separating two gratings with distinct Zak phases can support TIS mode. Because of the nonzero Zak phase difference between left and right gratings, a TIS exists in the nontrivial photonic bandgap when combining the left and right gratings, thereby enabling the TIS mode to extend uniformly across the cavity’s center. Figure 1C illustrates the simulated electrical field distribution with different *L*_TISE_/*L*. It is found that with larger *L*_TISE_/*L*, the TIS mode is expanded in the TISE cavity, improving the field uniformity.

At the cavity midpoint, a 150-μm radius MRR is integrated close to the TISE cavity, and the minimum gap between the MRR and the TISE laser is 300 nm. According to our calculations (see the “*Q* factor of MRR” section), for *R* > 150 μm, the increase in the *Q* factor is minimal, and further increasing *R* does not substantially improve the linewidth narrowing effect. As light propagates through the TISE laser, a portion of light is coupled into the MRR. After resonant circulation within the MRR, this light is reinjected into the TISE laser, achieving OIL. Figure 1 (D and E) shows the transmission spectrum and normalized photon distribution of the TISE laser compared against a conventional π-phase shift DFB laser. The results indicate that the TISE cavity produces a more uniform field distribution at the waveguide center. This uniformity increases the mode footprint and enhances the evanescent coupling efficiency between the TISE laser and the MRR. Figures 1F show the transmission spectrum from the MRR to the TISE laser and the quality factor *Q* = 1.1 × 10^5^. The efficiency of injection locking can be described by an equation derived for the reduction of the close-in linewidth of the laser (see the “Injection theory” section in the Supplementary Materials).

$$\delta \approx \frac{P_{r}}{P}\left(1+\alpha_{H}^{2}\right)\left(\frac{Q_{\text{MRR}}}{Q_{\text{LD}}}\right)$$ (1)

where *P_r_* is the feedback power from the MRR and *P* is the TISE laser power. *Q*_LD_ represents the quality factor of the laser, while *Q*_MRR_ denotes the quality factor of the MRR, α*_H_* is the linewidth enhancement factor. By substituting reasonable numbers in Eq. 1 for *P_r_/P* = 0.03, α*_H_* = 3, *Q*_MRR_ = 1.1 × 10^5^, and *Q*_LD_ = 4 × 10^3^, we find that the laser linewidth can be improved by a factor of 227. The locking range is defined as the frequency range over which the TISE laser emission injection locks to the MRR resonance and follows the expression

$$∆f_{\text{lock}}\approx \sqrt{\frac{P_{r}}{P}\left(1+\alpha_{H}^{2}\right)}\frac{f_{0}}{Q_{\text{LD}}}$$ (2)

where *f*_0_ represents the center frequency of the laser. The theoretically estimated locking range is $∆f_{\text{lock}}\approx 26\;\text{GHz}\;$.

**Fig. 2.**
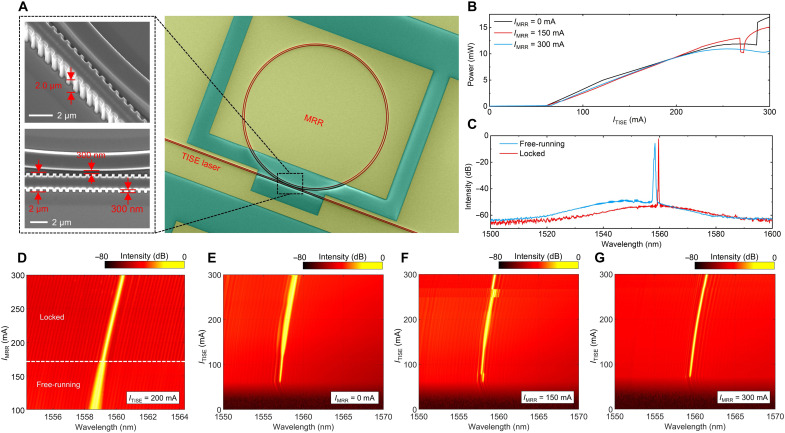
Structural imaging and optical spectrum narrowing. (**A**) SEM picture of the MOIL-TISE laser. (**B**) Typical *I*_TISE_*-P* characteristics with different *I*_MRR_. (**C**) Optical spectrum with the comparison of free-running (*I*_MRR_ = 0 mA) and locking state (*I*_MRR_ = 150 mA), *I*_TISE_ = 300 mA. (**D**) 2D spectrum as a function of *I*_MRR_ with *I*_TISE_ = 200 mA. (**E** to **G**) 2D spectrum as a function of *I*_TISE_ with *I*_MRR_ = 0 mA (E), 150 mA (F), and 300 mA (G), respectively.

The devices were fabricated using an AlGaInAs/InP epitaxial structure featuring five quantum wells (QWs) and six quantum barriers, with a QW confinement factor of 5% (*37*). The fabrication process of the MOIL-TISE laser is detailed in the “Fabrication process of MOIL-TISE laser” section in the Supplementary Materials. The room-temperature photoluminescence (PL) wavelength of the QWs was set to 1530 nm. A scanning electron microscope (SEM) image of the MOIL-TISE laser is shown in Fig. 2A. The minimum gap between the MRR and the TISE laser was 300 nm. The laser chip was mounted on a thermoelectric cooler set to 20°C to mitigate long-term drift. The MRR was also carrier injected to reduce the internal absorption loss of the MQW. Figure 2B shows the typical current-power (*I*_TISE_*-P*) characteristics of the TISE laser under different MRR injection currents (*I*_MRR_). The threshold current is ~65 mA, and the slope efficiency is about 0.07 W/A. Notably, varying *I*_MRR_ has only a minor influence on the TISE laser’s output power. At *I*_MRR_ = 300 mA, the saturation power is slightly reduced compared to *I*_MRR_ = 150 mA, likely due to the increased cavity temperature resulting from the higher injection current. Power skipping is observed in the *I*_*T*ISE_*-P* at *I*_MRR_ = 0 and 150 mA. This occurs because the TISE laser cannot achieve a stable locking state with the MRR when the MRR is not fully pumped, resulting in optical mode skipping. This phenomenon is also evident in the optical spectra shown in Fig. 2 (E to G). The laser is still able to deliver an output power up to 11 mW. Figure 2C compares the optical spectra of the laser in its injection-locked and free-running states. Under injection locking, the spectral linewidth is notably narrowed, and the SMSR exceeds 50 dB. To examine the locking state range, we fixed *I*_TISE_ at 200 mA and varied *I*_MRR_ from 100 to 300 mA, as shown in Fig. 2D. Once *I*_MRR_ surpasses 171 mA, the spectrum transitions from a broad (free-running) to a narrow (locked) linewidth, defining a locking range from 171 to 300 mA. The average current-induced wavelength red-shift coefficient (ACWRC) of the *I*_MRR_ is 0.01022 nm/mA. Figure 2 (E to G) presents two-dimensional (2D) spectral maps of the laser for *I*_*T*ISE_ values ranging from its threshold to 300 mA at *I*_MRR_  = 0, 150, and 300 mA. At *I*_MRR_ = 0 mA, the TISE laser and MRR are not locked, resulting in a broad spectral width. At *I*_MRR_ = 150 mA, the locking between the TISE laser and MRR is unstable, with longitudinal mode jitter observed at *I*_TISE_ = 77 and 260 mA, corresponding to the power skipping in Fig. 2B. At *I*_MRR_ = 300 mA, the TISE laser and MRR are fully locked, resulting in a narrow spectrum without skipping. The ACWRC for the *I*_TISE_ is 0.01038 nm/mA, indicating that the wavelength red-shift coefficient for TISE laser injection (with *I*_MRR_ fixed) closely matches that of MRR laser injection (with *I*_TISE_ fixed). As a result, the TISE laser and MRR exhibit excellent wavelength tuning synchronization, enabling a wide injection-locked range. These results confirm that the MOIL-TISE laser remains in the injection-locked state over a wide current range, maintaining stable locking for *I*_TISE_ from threshold 65 to 300 mA when *I*_MRR_ = 300 mA.

**Fig. 3.**
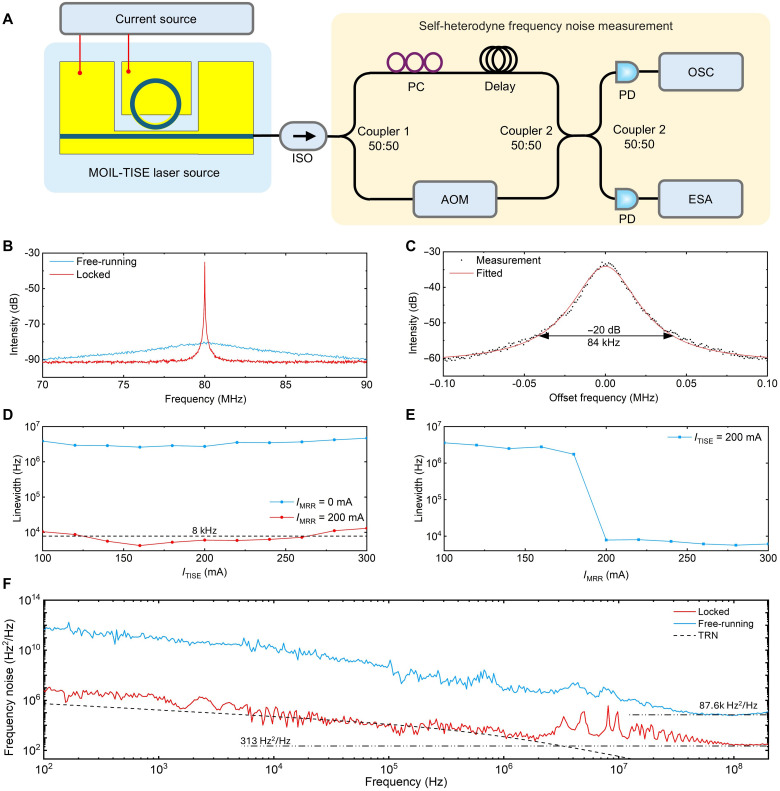
Laser linewidth and frequency noise measurement. (**A**) Experimental setup: Light is collected from the TISE facet with a lensed fiber, and a nonzero frequency self-heterodyne setup is used to measure the linewidth. ISO, isolator; AOM, acoustic-optic modulator; PC, polarization controller; PD, photodetector; ESA, electrical spectrum analyzer; OSC, oscilloscope. (**B**) Measured radio frequency signal from PD with injecting locked *I*_MRR_ = 200 mA and *I*_TISE_ = 160 mA, yielding a dramatic narrowing of the emission linewidth compared to the free-running regime (*I*_MRR_ = 0 mA). (**C**) Voigt-fitted linewidth with *I*_MRR_ = 200 mA and *I*_TISE_ = 160 mA. (**D**) Linewidth as a function of *I*_TISE_ with *I*_MRR_ = 0 and 200 mA. (**E**) Linewidth as a function of *I*_MRR_ with *I*_TISE_ = 200 mA. (**F**) Noise spectra of laser measured by correlated DSH measurement between locked (*I*_MRR_ = 160 mA and *I*_TISE_ = 200 mA) and free-running signal (*I*_MRR_ = 0 mA and *I*_TISE_ = 200 mA). The dotted blue line indicates the calculated TRN limit for MRR.

We then investigated the coherence properties of the MOIL-TISE laser. The laser linewidth was measured using the fiber-delayed, nonzero frequency delayed self-heterodyne (DSH) method, using a 25-km single-mode fiber delay and an 80-MHz acoustic-optic modulator (AOM), as shown in Fig. 3A. A comparison of the free-running and injection-locked states is presented in Fig. 3B, revealing a pronounced linewidth narrowing under injection locking. A Voigt fitting of the spectrum is shown in Fig. 3C. Because Gaussian noise has a more prominent effect on broadening the spectral line near the center (*38*, *39*), the intrinsic Lorentzian linewidth was estimated by measuring the −20-dB bandwidth and applying a $2\sqrt{99}$ division factor to reduce the effect of Gaussian noise (*40*). Stable injection locking is obtained with an intrinsic Lorentzian linewidth of 4.2 kHz. We further examined the linewidth variation as a function of *I*_TISE_ and compared it to the free-running state, as illustrated in Fig. 3D. Under free-running conditions, the intrinsic linewidth ranged from 1.7 to 3.6 MHz, whereas injection locking reduced it to 4.2 to 13 kHz—an improvement by a factor greater than 250, which corresponds to the calculated reduction of the close-in linewidth factor. The linewidth is <8 kHz within the *I*_TISE_ range of 140 to 260 mA. Figure 3E shows the linewidth reduction as *I*_MRR_ increases, with the laser transitioning from the free-running to the locked state. The thermomechanical drift of the resonator introduces additional linewidth broadening effects; therefore, the spectral purity of the laser cannot be fully characterized by a purely Gaussian or Lorentzian line shape. To confirm the linewidth, we used the correlated DSH FN-PSD method. The recorded frequency noise is displayed in Fig. 3F. A white noise floor of ~313 Hz^2^/Hz is observed, which corresponds to an intrinsic linewidth (Lorentzian linewidth) of 983 Hz for the laser. This further confirms the narrow-linewidth performance of the laser in the locked state, in contrast to the free-running state, where the intrinsic linewidth is 275.2 kHz. We calculate the thermo-refractive noise (TRN) of the MRR (Materials and Methods), the PSD under injection locked condition is in good agreement with fundamental TRN in 10 kHz to 1 MHz, indicating that the laser performance may ultimately be limited by TRN in this frequency range.

**Fig. 4.**
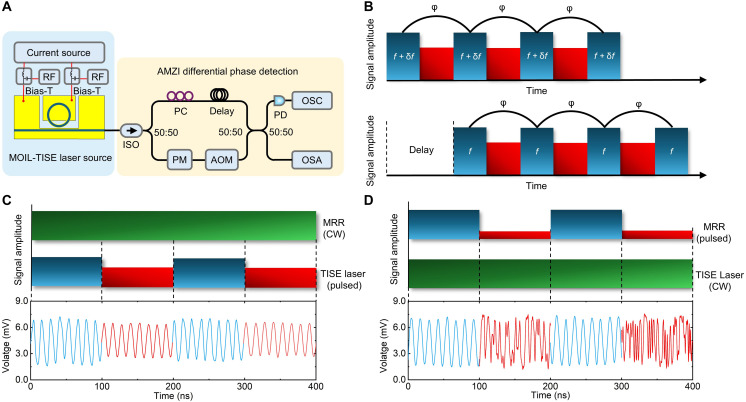
Injection locking phase encoding based on MOIL-TISE laser. (**A**) Experimental setup of the AMZI; PM, phase modulator. (**B**) Principle of the operation of the phase detection. (**C**) When the MRR operates in CW and is injected into the TISE laser, the TISE laser pulses all inherit the coherence. (**D**) When the TISE operates in CW mode and the MRR switches between locked and free-running states, the TISE laser pulses transition between phase-locked and random states.

At the heart of the QKD transmitter, the quantum state encoding engine must be capable of encoding or randomizing multiple phase states, either with deterministic phase values or driven by high-entropy random numbers. OIL has been proposed as a technique for realizing a modulator-free QKD transmitter chip (*41*). This approach leverages the combination of injection locking and ultrafast phase modulation between the master and slave lasers to generate chirp-free optical pulses and enable multilevel phase encoding, eliminating the need for high-speed external modulators (*42*). For the MOIL-TISE laser, we evaluated the coherence of successive pulses in a pulse train under two optical injection conditions: a continuous wave (CW) MRR and a directly modulated MRR. The measurement setup, depicted in Fig. 4A, is based on an asymmetric Mach-Zehnder interferometer (AMZI). A thermal phase modulator (PM) in the short arm controls the relative phase between the two arms, while an AOM introduces an 80-MHz frequency shift. This shift facilitates the differentiation of low-frequency electronic noise from the beat signal on the PD, thus improving the measurement accuracy and signal-to-noise ratio. The TISE laser was driven by a 5-MHz radio frequency signal with voltage of peak to peak at 400 mV, and the AMZI was configured with a 200-ns delay between its short and long arms—matching the 200-ns interval between consecutive pulses at 5 MHz (Fig. 4B). Under the CW-locked MRR condition, as illustrated in Fig. 4C, each pulse inherits coherence from the locking process, and the phase difference between consecutive pulses remains fixed. This behavior closely mimics the saturation regime of the COW protocol. Conversely, when the MRR is directly modulated and the TISE laser operates in CW mode, the MOIL-TISE laser cycles between phase-locked and random-phase states, causing the phase of consecutive pulses to vary and no longer remain fixed in Fig. 4D.

## DISCUSSION

The concept of injection locking—recently used in narrow-linewidth semiconductor lasers through heterogeneous integration of III to V gain media and ultrahigh-Q silicon-based microresonators—has been extensively explored for coherent optical systems. In contrast to heterogeneous approaches, monolithic integration unifies the material platform, reducing the device footprint, fabrication complexity, and cost. However, most monolithic π-phase–shifted DFB lasers exhibit linewidths of megahertz. In this work, we present a monolithically integrated MOIL-TISE laser fabricated on an InP platform. Our measurements show that the MOIL-TISE laser achieves a Voigt-fitted linewidth of 4.2 kHz and an intrinsic linewidth of 983 Hz, as determined by FN-PSD. Table 1 compares the performance of our MOIL-TISE lasers with various hybrid and monolithic III-V narrow-linewidth semiconductor lasers. The linewidth results obtained from the FN-PSD and DSH methods differ, with FN-PSD generally considered more reliable. Therefore, both values are listed separately in Table 1 according to their respective measurement methods. While the MOIL-TISE laser shows a performance gap compared to state-of-the-art hybrid ECL/SIL systems using ultrahigh-Q external resonators (*10*, *43*, *44*), it achieves the narrowest linewidth among monolithic InP single-chip DFB lasers operating at 1550 nm. Its performance is comparable to some hybrid SIL/ECL devices, demonstrating its competitive potential.

**Table 1.** Performance comparison. The performances of hybrid integrated SIL, ECL lasers, conventional DFB, and DBR lasers operating at a wavelength of 1550 nm.

Reference	Structure	Power (mW)	SMSR (dB)	Linewidth (kHz)
(*22*) (2018)	Si-Hybrid ECL	11	>46	37 (FN-PSD)
(*47*) (2021)	Si-Hybrid ECL	>2	>40	105 (FN-PSD)
(*48*) (2024)	Si-Hybrid ECL	16.4	>45	2.79 (DSH)
(*49*) (2025)	Si-Hybrid ECL	10	40	1.6 (FN-PSD)
(*21*) (2022)	Si-Hybrid SIL	>2	>40	27 (FN-PSD)
(*23*) (2023)	Si-Hybrid ECL	76	>50	12 (FN-PSD)
(*50*) (2020)	SiN-Hybrid ECL	0.5	>55	4 (FN-PSD)
(*51*) (2021)	SiN-Hybrid SIL	37.9	56	3 (FN-PSD)
(*43*) (2025)	SiN-Hybrid ECL	>4	65	(3–7) ×10^−3^ (FN-PSD)
(*10*) (2021)	SiN-Hybrid SIL	/	/	1.2 × 10^−3^ (FN-PSD)
(*44*) (2025)	Vacuum FP cavity	/	/	3.5 × 10^−5^ (FN-PSD)
(*25*) (2022)	LNOI-Hybrid ECL	3.7	>50	15 (DSH)/11.3 (FN-PSD)
(*52*) (2024)	LNOI-Hybrid SIL	0.74	/	45.5 (DSH)
(*53*) (2024)	LNOI-Hybrid SIL	3.18	60	2.5 (FN-PSD)
(*54*) (2025)	LNOI-Hybrid ECL	15	63	2.8 (FN-PSD)
(*55*) (2015)	InP-DBR Butt joint	>120	>50	70 (FN-PSD)
(*56*) (2022)	InP-QW DFB	45	58.7	63 (DSH)
(*57*) (2022)	InP-DBR Butt joint	18	54	45 (DSH)/10 (FN-PSD)
(*58*) (2019)	InP-QW DFB	/	/	100 (DSH)
This work	InP MOIL-TISE	11	>50	4.2 (DSH)/0.983 (FN-PSD)

In this work, we present a single-chip MOIL-TISE laser fabricated on an InP platform. This device combines an MRR with a quality factor of 10^5^ and a TISE laser. The TISE laser uses a mode-extension cavity that expands the fundamental optical mode from the cavity center to its edges, producing a uniform photon density distribution at the cavity center, thereby enhancing its coupling efficiency to the MRR. Our measurements show that the injection-locked MOIL-TISE laser achieves a Voigt-fitted linewidth of 4.2 kHz, FN-PSD linewidth of 983 Hz, SMSR of >50 dB, and a wide current locking range of 65 to 300 mA. The fabrication process of the MOIL-TISE laser is also the simplest, with only one step of MOVPE epitaxial growth and one step of ICP dry etch. In addition, our experiments demonstrate the intrinsic phase-adjustment capability of the MOIL-TISE laser, highlighting its potential for use in both quantum and conventional coherent communication systems. This intrinsic phase control also enables the seamless combination of QKD and high-bandwidth data transmission, underscoring the versatility and promise of the MOIL-TISE laser platform.

## MATERIALS AND METHODS

### Device fabrication

The fabrication procedure for the HF-OIL laser can be found in the “Fabrication process of MOIL-TISE laser” section in the Supplementary Materials. The wafer was grown on an InP substrate using MOVPE. The room-temperature PL peak of the QWs was located at a wavelength of 1530 nm. The TISE laser sidewall grating and MRR waveguide was defined by electron-beam lithography (EBL) on an EBPG5200 E-beam system, with negative-tone hydrogen silsesquioxane (HSQ) acting as both the EBL resist and a hard mask for ICP dry etching using a Cl_2_/CH_4_/H_2_ and CH_4_/H_2_/O_2_ gas mixture in an Oxford PlasmaPro 300 system. Subsequent steps included plasma-enhanced chemical vapor deposition (PECVD) deposition of SiO_2_ (Oxford PlasmaPro 100 PECVD), application of HSQ passivation layers, SiO_2_ window opening, P-contact deposition, substrate thinning, and N-contact deposition, all performed using conventional laser diode fabrication techniques. These two microelectrodes only require one step of lithography, together with the large area electrode pads. SEM images were acquired using a Hitachi SU8240 SEM operating at 10 kV.

### Thermo-refractive noise

Continuous heat exchange between the microresonator and its surrounding environment leads to thermodynamic fluctuations, which, through the thermo-optic effect, induce variations in the refractive index. These fluctuations manifest as TRN in the resonant frequencies (*45*, *46*). The variance of the TRN is expressed as

$$\begin{array}{c} S_{\text{TRN}}\left(\omega \right)={\left(f_{0}\frac{1}{n_{0}}\frac{\mathrm{dn}}{\mathrm{dT}}\right)}^{2}S_{\delta T}\left(\omega \right)\\ S_{\delta T}\left(\omega \right)=\frac{k_{B}T^{2}}{\sqrt{\pi^{3}\kappa \rho C\omega }}\sqrt{\frac{1}{2p+1}}\frac{1}{R\sqrt{d_{x}^{2}-d_{y}^{2}}}\frac{1}{{\left[1+{\left({\omega \tau }_{d}\right)}^{3/4}\right]}^{2}}\\ \tau_{d}={\left(\frac{\pi }{4}\right)}^{1/3}\frac{\rho c}{\kappa }d_{x}^{2} \end{array}$$ (3)

Where *n*_0_ is the refractive index, *k*_B_ is the Boltzmann constant, and *f*_0_ is the resonant frequency. *T* is the temperature; κ is the thermal conductivity; ρ is the material density; *C* is the heat capacity, ω is the response frequency; *d_x_* and *d_y_* are the halfwidths of the eigenmode along the *x* and *y*directions, respectively; *p* is the meridional mode number; and *dn*/*dT* is the thermo-optic coefficient. *R* is the MRR radius.

### Measurement setup

The laser was driven by a continuous-wave current source (Newport Model 8000). An isolator was placed at the laser output. The light passed through the isolator and entered coupler 1, which had a splitting ratio of 50:50, dividing the light into two paths. One path included a time-delay fiber and a polarization controller (FPC032), while the other path included an 80-MHz AOM (AeroDIODE, 1550-AOM). These two paths were then combined in coupler 2 and split again into two paths: one leading to a photodetector (Thorlabs, DET08CFC) connected to an electronic spectrum analyzer (Keysight, N9000B) and the other to an oscilloscope. Both the current driver and the electronic spectrum analyzer, as well as the oscilloscope, were controlled via a General Purpose Interface Bus using LabVIEW software. For the correlated DSH FN-PSD method, the experimental setup is similar to the one shown in Fig. 3A, with the key difference being a 40-m-long fiber line to delay the optical path. In the phase encoding experiment, a phase modulator PM (LNP6118) was used to control the relative phase between the two AMZI arms.

## Supplementary Materials

### This PDF file includes:

Supplementary Text Figs. S1 to S4 References
