# Supplementary material for: Narrow-linewidth monolithic topological interface state extended laser with optical injection locking
Source: Sci Adv. 2025 Sep 10;11(37):eady8963. doi: 10.1126/sciadv.ady8963 (PMC12422184; doi:10.1126/sciadv.ady8963)
Supplement: Supplementary file 1 — Supplementary Text Figs. S1 to S4 References [file sciadv.ady8963_sm.pdf]

Science Advances
AAAS

# Supplementary Materials for

## Narrow-linewidth monolithic topological interface state extended laser with optical injection locking

Xiao Sun *et al.*

Corresponding author: Xiao Sun, xiao.sun@glasgow.ac.uk; Lianping Hou, lianping.hou@glasgow.ac.uk

*Sci. Adv.* **11**, eady8963 (2025)
DOI: 10.1126/sciadv.ady8963

**This PDF file includes:**

Supplementary Text
Figs. S1 to S4
References

## Supplementary Text

### Supplementary Part A: Comparison of uniform and sampling grating modulations in TISE cavity

Fig. S1 compares the simulated photon density distribution of uniform grating modulation and sampling grating modulation. The uniform grating is designed with a third-order grating with period at $\Lambda_B$ = 720 nm, the modulation period is $\Lambda_M = \Lambda_B \times 5$ = 3.6 µm, with a shift step $\Lambda_B/5$ = 144 nm. The TISE cavity consists of 28 modulation periods ($\Lambda_M$) to realize a total TISE cavity length of 100 µm. In the Sampling grating modulation, we set the sample grating period $\Lambda_S$ = 3.125 µm, modulation period $\Lambda_M = \Lambda_S \times 8$ = 25 µm. The TISE cavity, in this case, also consists of four modulation periods ($\Lambda_M$) for a total TISE length of 100 µm. The total cavity length is 1000 µm, and the photon density distribution is calculated by the coupling-wave method with a transfer matrix. It can be found that the uniform grating modulation has better photon uniformity in the TISE cavity. Given that our E-beam lithography system (EBPG5200) has a resolution of 0.5 nm, direct modulation of the Bragg uniform grating is feasible from a fabrication perspective.

### Supplementary Part B: Q factor of MRR

We investigated the effect of loss on the MRR quality factor ($Q$), Figure. S2(A) presents the cross-section schematic of the MRR and laser. The $Q$ of the InP MQW MRR is given as (*59, 60*):

$$Q \cong \frac{2\pi n_v R}{\lambda} \frac{\pi \sqrt[4]{1-\eta_c} e^{(-\alpha_i)\pi R/2}}{1-\sqrt[2]{1-\eta_c} e^{(-\alpha_i)\pi R}} \tag{S1}$$

Where $n_v$ is the group index, $R$ is the radius of the MRR, $\alpha_i$ is the internal loss, $\lambda$ is the longitudinal mode wavelength, $n_c$ is the coupling factor, which is calculated from the difference in index($\Delta n$) between the two coupled modes ( Mode 1 and Mode 2), as shown in Fig. S2(B).

$$\eta_c = \sin^2\left(\frac{\pi \Delta n L_c}{\lambda}\right) \tag{S2}$$

Where $L_c$ is the coupling length. We fixed the ridge waveguide width ($W$) of the MRR and laser at 2 µm. Figure S2(C) illustrates the $\eta_c$ as a function of gap width, where $R$ is fixed at 150 µm, so $L_c$ is ~18 µm. For the gap width = 300 nm, the $\eta_c$ value is about 0.03. The precise control of $\eta_c$, via accurate control of the gap size, can be achieved by nanofabrication technology, as has been demonstrated with InP materials. The $Q$ factor as a function of $R$ is shown in Figure S2(D). It can be observed that for $R$ > 150µm, the increase in the $Q$ factor is minimal. As a result, further increasing $R$ does not significantly improve the linewidth narrowing effect. The internal loss ($\alpha_i$) is mainly decided by the bending radiation loss ($\alpha_{rad}$) and material absorption loss ($\alpha_m$=15/cm). The bending radiation loss is affected by bending radius ($R$), ridge width ($W$), and ridge height ($H$). In the fabrication procedure, the gap and ridge width can be precisely defined by e-beam lithography. The ridge height is controlled by the $CH_4/H_2/O_2$ chemistry, in which the Al-containing layer can be used to stop near the top of the QWs. The etching process is stopped approximately 40 nm above the MQW layer, with $H$ = 2 µm. Therefore the $\alpha_m$ is calculated about 0.7/cm for $R$ = 150 µm, $W$= 2 µm and $H$ = 2 µm (*61*). Figure S2(E) illustrates the calculated $Q$ as a function of gap width (line); here we only consider the $\alpha_{rad}$ = 0.7/cm as the internal loss and agree with the

FDTD simulation result (dot). The material loss can be balanced by the current injection to the MQW.

## Supplementary Part C: Injection theory

In this section, we apply the theory of optical injection locking (OIL), which is a modification of the semiclassical laser rate equations by introducing additional terms of light intensity (or the number of photons) and phase offset to describe the impact of the laser injection signal. In this analysis, we make a series of simplifying assumptions. Specifically, unlike the self-injection locking (SIL) model that relies on backscattering feedback, our laser is directly coupled to the MRR. We ignore all nonlinearities coming from the longitudinal modes propagating in the opposite direction other than the pump mode and consider that the backscattering is zero. Also, we do not include the semiconductor gain dynamics in the analysis. Instead, we assume that the gain adiabatically follows the pump power since the semiconductor dynamics are much faster than the cavity mode dynamics. For the semiconductor laser with the optical injection feedback, the laser system can be described by the Lang-Kobayashi equation as follows (*62 ,63*), which describe the relation between carrier number $N(t)$, the photon number $S(t)$ and optical phase $\phi(t)$:

$$\begin{aligned}
\frac{dS(t)}{dt} &= g\frac{N(t)-N_0}{1+\varepsilon S(t)}S(t) - \frac{S(t)}{\tau_p} + \frac{\beta N(t)}{\tau_c} + F_{S(t)} \\
\frac{dN(t)}{dt} &= \frac{I(t)}{q} - \frac{N(t)}{\tau_c} - g\frac{N(t)-N_0}{1+\varepsilon S(t)}S(t) + F_{N(t)} \\
\frac{d\phi(t)}{dt} &= \frac{\alpha}{2}g(N(t)-\overline{N}) + F_{\phi(t)}
\end{aligned} \tag{S3}$$

where $I(t)$ is the injection current, $q$ is the electron charge, $\tau_c$ is the carrier lifetime, $\tau_p$ is the photon lifetime, $g$ is the differential gain coefficient, $\varepsilon$ is the nonlinear gain compression factor, $N_0$ is the carrier number at transparency, $\beta$ is the fraction of spontaneous emission coupled into the lasing mode, $\overline{N}$ is the time-averaged carrier number. $F_{S(t)}$, $F_{N(t)}$, and $F_{\phi(t)}$ are the Langevin forces for the relative photon, carrier, and phase. If only noise from spontaneous emissions is present, we have diffusion coefficients given by:

$$\begin{aligned}
\langle F_i(t)\rangle &= 0 \\
\langle F_i(t)F_j(t')\rangle &= 2D_{ij}\delta(t-t')
\end{aligned} \tag{S4}$$

where $\delta$ is the Dirac's delta function and $D_{i,j}$ is the diffusion coefficient associated with the corresponding noise source defined as

$$\begin{aligned}
D_{SS} &= \frac{\beta N(t)S(t)}{\tau_c} \\
D_{NN} &= \frac{N(t)}{\tau_c}[1+\beta S(t)] \\
D_{\phi\phi} &= \frac{\beta N(t)}{4\tau_c S(t)} \\
D_{S\phi} &= D_{N\phi} = 0
\end{aligned} \tag{S5}$$

The intrinsic Lorentz linewidth formula of the single-mode laser can be obtained:

$$\begin{aligned}
\Delta\nu_0 &= \frac{R_{sp}}{S}(1+\alpha_H{}^2) \\
R_{sp} &= \beta\eta_{sp}(A+BN+CN^2)N
\end{aligned} \tag{S6}$$

Where $R_{sp}$ is the spontaneous emission rate, $\eta_{sp}$ is the spontaneous quantum efficiency, and $\alpha_H$ is the linewidth enhancement factor.
The feedback field $S(t)$ from the MRR can be described by:

$$S'(t) = \frac{P_r}{P} S(t-\tau) \tag{S7}$$

Where $P_r$ is the feedback power from the MRR $P$ is the TISE laser power, consider the steady-state solution. The feedback field $S(t)$ from the MRR can be described as:

$$\begin{aligned} S'(t) &= T(\omega)^2 \cdot S(t-\tau) \\ T(\omega) &= \sqrt{\frac{P_r}{P}} \cdot e^{i\omega\tau_R} \end{aligned} \tag{S8}$$

Where $\omega$ is the free-running laser frequency, $\tau_R$ is the round-trip time in the MRR. When the TISE laser and MRR are injected locking, the system is in a steady state. The Equation (S3) for the steady-state solution is:

$$\begin{aligned} & g\frac{N_s - N_0}{1+\varepsilon S_s} S_s - \frac{S_s}{\tau_p} + \frac{\beta N_s}{\tau_c} + \kappa \operatorname{Re}[T(\omega_s)] = 0 \\ & \frac{I_s}{q} - \frac{N_s}{\tau_c} - g\frac{N_s - N_0}{1+\varepsilon N_s} S_s = 0 \\ & \omega_0 - \omega_s + \frac{\alpha}{2} g(N_s - \overline{N}) - \kappa \operatorname{Im}[T(\omega_s)] = 0 \end{aligned} \tag{S9}$$

$N_s$ , $S_s$ and $\omega_s$ are the steady-state solutions. $\omega_0$ are the TISE laser wavelength without feedback. $\kappa$ is the coupling coefficient of the TISE grating. The linewidth reduction factor $\delta$ at low offset frequencies can be calculated using

$$\delta = \left( \frac{\partial(\Delta\omega_0)}{\partial(\Delta\omega)} \right)^2 \tag{S10}$$

The maximum $\delta$ occurs at $\omega * \tau_R$ = -arctanh($\alpha_H$) + $2n\pi$ (n is integer):

$$\delta \approx \frac{P_r}{P}(1+\alpha_H^2)\left( \frac{Q_{MRR}}{Q_{LD}} \right)^2 \tag{S11}$$

$Q_{LD/MRR}$ is the quality factor of laser or MRR. Since the coupling coefficient is inversely proportional to the quality factor $Q$, a laser with low $Q$ will have a larger locking range than lasers with high $Q$. Therefore, low $Q$ lasers are easier to lock. However, low $Q$ also leads to increased laser linewidth which reduces the phase noise performance of the OIL laser system. The injection locking bandwidth is given as (*64*):

$$\sigma_o = \sigma_f + \frac{3K}{2} \times \frac{2\sigma_f \cos\varphi + (1-\sigma_f^2)\sin\varphi}{(1-\sigma_f^2)^2 + 4\sigma_f^2} \tag{S12}$$

Where $\sigma_o$ is the output frequency detuning and $\sigma_f$ is the free-running frequency detuning, $\sigma_{o/f} = 2\Delta f_{o/f} Q_{MRR} / f_0$ . $K$ is the injection ratio and $K = \sqrt{\delta}$ , $\varphi$ is the locking phase is delayed. A high injection ratio results in a large locking range, which also makes the laser easier to lock. The locking range of the frequency is defined as:

$$\Delta f_{lock} \approx \sqrt{\frac{P_r}{P}(1+\alpha_H^2)} \frac{f_0}{Q_{LD}} \tag{S13}$$

The injection locking bandwidth curve is shown in Fig. S3, where $Q_{MRR} = 1.1\times10^5$, $Q_{LD} = 5\times10^3$. Fig. S3(C) presents an optimal injection locking of the MRR and TISE laser with $\varphi = 0$ and $K$ = 15. The locking range is 26.3 GHz.

**Supplementary Part D: Fabrication process of MOIL-TISE laser**

The fabrication procedure for the MOIL-TISE laser is shown in Fig. S4. The wafer was grown on an InP substrate using metalorganic vapor-phase epitaxy (MOVPE). The 1D-TISE-PhC sidewall grating waveguide was defined by electron-beam lithography (EBL) on an EBPG5200 E-beam system with a dose at 800 μC/cm$^2$, with negative-tone Hydrogen Silsesquioxane (HSQ) acting as both the EBL resist and a hard mask for inductively coupled plasma (ICP) dry etching using a $Cl_2/CH_4/H_2$ (6/12/10 sccm) gas mixture in an Oxford PlasmaPro 300 system with RF/ICP power at 150W/500W. When the etch depth reached approximately 1.9 μm, the ICP recipe was switched to a $CH_4/H_2/O_2$ gas mixture (6/40/0.2 sccm) with RF/ICP power set to 200 W/100 W to terminate etching at the Al-containing layer near the top of the QWs (about 2.0 μm depth). Subsequent steps included PECVD deposition of $SiO_2$ (Oxford PlasmaPro 100 PECVD) at 300°C, application of HSQ planarization layers, $SiO_2$ window opening, P-contact deposition (Ti/Pt/Au), substrate thinning, and N-contact deposition (Au/Ge/Au/Ni/Au), all performed using conventional laser diode (LD) fabrication techniques. Scanning electron microscopy (SEM) images were acquired using a Hitachi SU8240 scanning electron microscope operating at 10 kV.

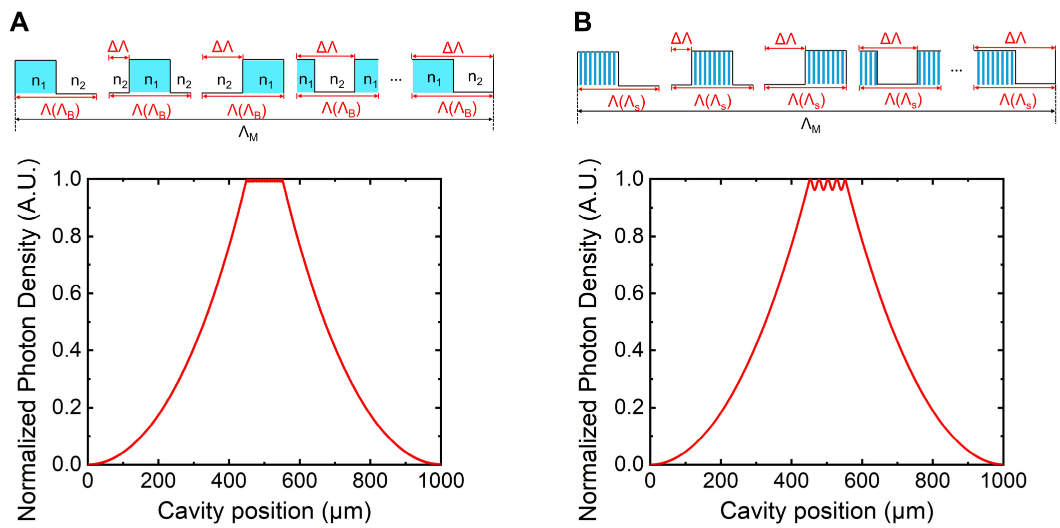


**Fig. S1. Comparison of uniform and sampling grating modulations.** (**A**) uniform grating modulation and the corresponding photon density distribution; $\Lambda(\Lambda_B)$ = 720 nm, $\Lambda_M$ = 3.6 µm. The TISE cavity consists of 28×$\Lambda_M$. (**B**) Sampling grating modulation and the corresponding photon density distribution; $\Lambda(\Lambda_S)$ = 3.125 µm, $\Lambda_M$ = 25 µm. The TISE cavity consists of 4 ×$\Lambda_M$.

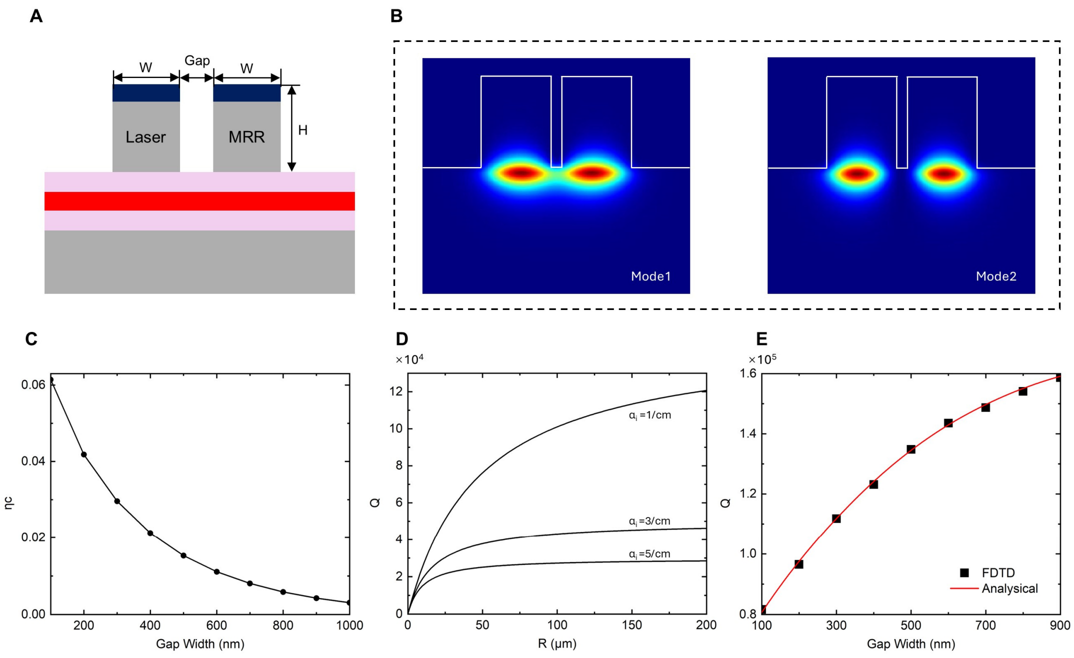


**Fig. S2. Optical modes and Q factor calculation.** (**A**) Cross-section schematic of the MRR and laser. (**B**) Electrical field distribution of two coupled modes with gap width at 300 nm. (**C**) Calculated coupling factor as a function of gap width and $R$ = 150 μm. (**D**) Calculated $Q$ factor as a function of $R$ under different losses, the gap width is 300 nm. (**E**) Calculated $Q$ factor as a function of gap width compared with the FDTD simulated result, with $R$ = 150 μm and $\alpha_i = 0.7$/cm.

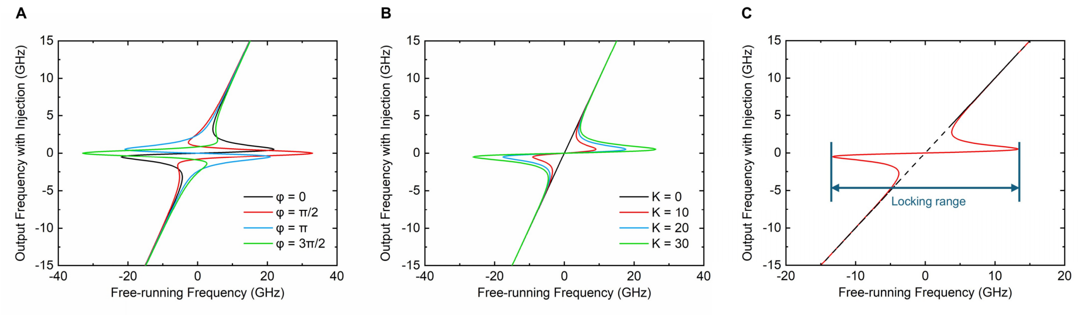


**Fig. S3. Injection locking bandwidth.** (**A**) bandwidth curve with $\varphi = 0, \pi/2, \pi, 3\pi/2$, $K = 25$. (**B**) curve with $\varphi = 0$, $K = 0$, 10, 20, 30. (**C**) Optimal injection locking of MRR and TISE laser with $\varphi = 0$ and $K = 15$.

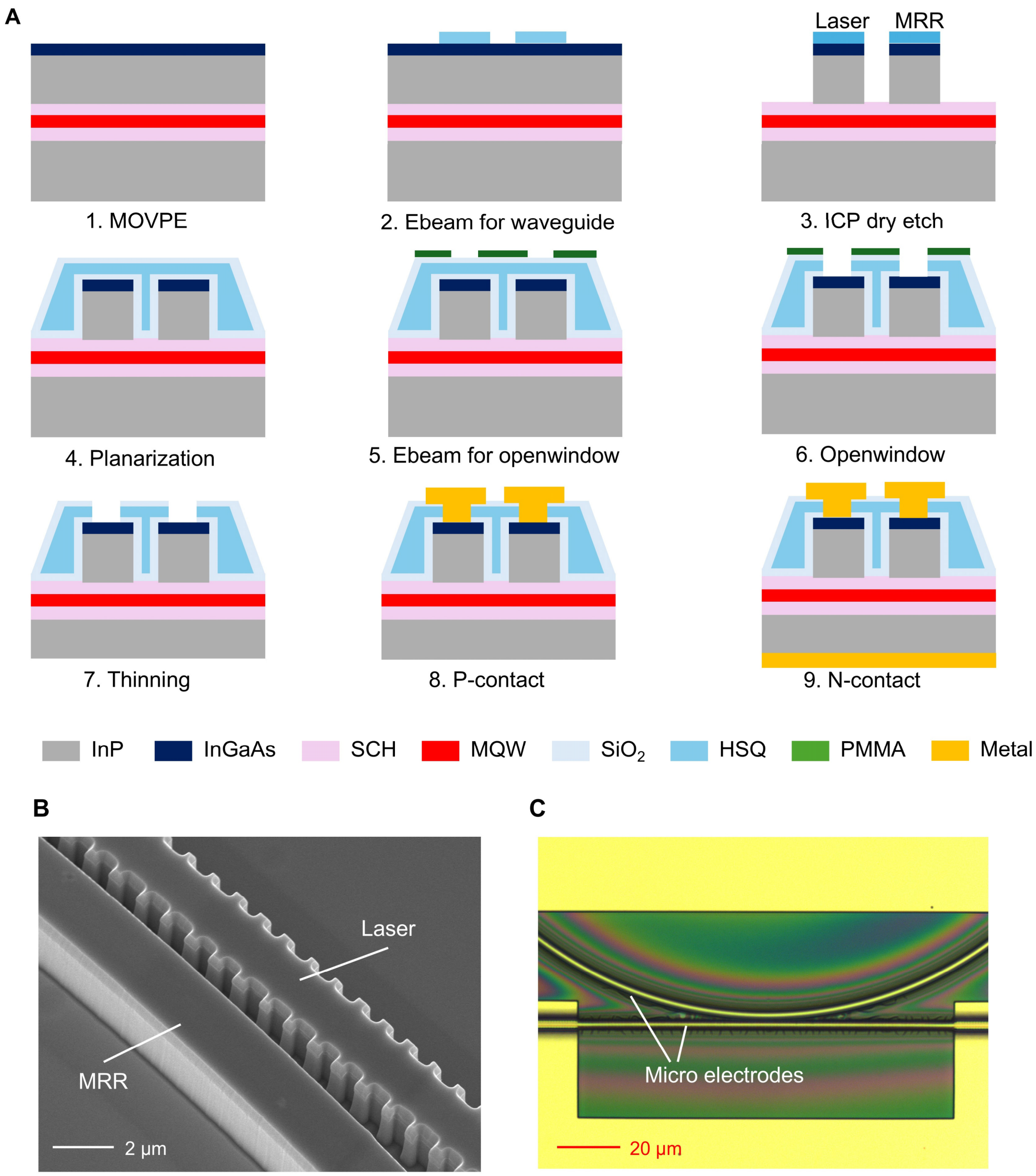


**Fig. S4. MOIL-TISE laser fabrication.** (**A**) Fabrication process of MOIL-TISE laser. (**B**) SEM image of the MRR and TISE laser coupling range. (**C**) Microscope image of the electrode on the MRR and TISE laser.

## REFERENCES AND NOTES


1. E. Luvsandamdin, C. Kürbis, M. Schiemangk, A. Sahm, A. Wicht, A. Peters, G. Erbert, G. Tränkle, Micro-integrated extended cavity diode lasers for precision potassium spectroscopy in space. *Opt. Express* **22**, 7790–7798 (2014).

2. M.-G. Suh, Q.-F. Yang, K. Y. Yang, X. Yi, K. J. Vahala, Microresonator soliton dual-comb spectroscopy. *Science* **354**, 600–603 (2016).

3. H. Katori, Optical lattice clocks and quantum metrology. *Nat. Photonics* **5**, 203–210 (2011).

4. Z. L. Newman, V. Maurice, T. Drake, J. R. Stone, T. C. Briles, D. T. Spencer, C. Fredrick, Q. Li, D. Westly, B. R. Ilic, B. Shen, M.-G. Suh, K. Y. Yang, C. Johnson, D. M. S. Johnson, L. Hollberg, K. J. Vahala, K. Srinivasan, S. A. Diddams, J. Kitching, S. B. Papp, M. T. Hummon, Architecture for the photonic integration of an optical atomic clock. *Optica* **6**, 680–685 (2019).

5. P. Trocha, M. Karpov, D. Ganin, M. H. P. Pfeiffer, A. Kordts, S. Wolf, J. Krockenberger, P. Marin-Palomo, C. Weimann, S. Randel, W. Freude, T. J. Kippenberg, C. Koos, Ultrafast optical ranging using microresonator soliton frequency combs. *Science* **359**, 887–891 (2018).

6. M.-G. Suh, K. J. Vahala, Soliton microcomb range measurement. *Science* **359**, 884–887 (2018).

7. J. Liu, E. Lucas, A. S. Raja, J. He, J. Riemensberger, R. N. Wang, M. Karpov, H. Guo, R. Bouchand, T. J. Kippenberg, Photonic microwave generation in the X-and K-band using integrated soliton microcombs. *Nat. Photonics* **14**, 486–491 (2020).

8. D. Marpaung, J. Yao, J. Capmany, Integrated microwave photonics. *Nat. Photonics* **13**, 80–90 (2019).

9. W. Liang, D. Eliyahu, V. S. Ilchenko, A. A. Savchenkov, A. B. Matsko, D. Seidel, L. Maleki, High spectral purity Kerr frequency comb radio frequency photonic oscillator. *Nat. Commun.* **6**, 7957 (2015).

10. W. Jin, Q.-F. Yang, L. Chang, B. Shen, H. Wang, M. A. Leal, L. Wu, M. Gao, A. Feshali, M. Paniccia, K. J. Vahala, J. E. Bowers, Hertz-linewidth semiconductor lasers using CMOS-ready ultra-high-Q microresonators. *Nat. Photonics* **15**, 346–353 (2021).

11. C. Xiang, W. Jin, O. Terra, B. Dong, H. Wang, L. Wu, J. Guo, T. J. Morin, E. Hughes, J. Peters, Q.-X. Ji, A. Feshali, M. Paniccia, K. J. Vahala, J. E. Bowers, 3D integration enables ultralow-noise isolator-free lasers in silicon photonics. *Nature* **620**, 78–85 (2023).

12. J. Guo, C. A. McLemore, C. Xiang, D. Lee, L. Wu, W. Jin, M. Kelleher, N. Jin, D. Mason, L. Chang, A. Feshali, M. Paniccia, P. T. Rakich, K. J. Vahala, S. A. Diddams, F. Quinlan, J. E. Bowers, Chip-based laser with 1-hertz integrated linewidth. *Sci. Adv.* **8**, eabp9006 (2022).

13. Y. Fan, A. van Rees, P. J. Van der Slot, J. Mak, R. M. Oldenbeuving, M. Hoekman, D. Geskus, C. G. Roeloffzen, K.-J. Boller, Hybrid integrated InP-$Si_3N_4$ diode laser with a 40-Hz intrinsic linewidth. *Opt. Express* **28**, 21713–21728 (2020).

14. C. Xiang, W. Jin, J. E. Bowers, Silicon nitride passive and active photonic integrated circuits: Trends and prospects. *Photonics Res.* **10**, A82–A96 (2022).

15. S. Qiao, Y. He, H. Sun, P. Patimisco, A. Sampaolo, V. Spagnolo, Y. Ma, Ultra-highly sensitive dual gases detection based on photoacoustic spectroscopy by exploiting a long-wave, high-power, wide-tunable, single-longitudinal-mode solid-state laser. *Light Sci. Appl.* **13**, 100 (2024).

16. Q. Ruan, X. Xiao, J. Zou, H. Wang, S. Fan, T. Li, J. Li, Z. Dong, Z. Cai, Z. Luo, Visible-wavelength spatiotemporal mode-locked fiber laser delivering 9 ps, 4 nJ pulses at 635 nm. *Laser Photonics Rev.* **16**, 2100678 (2022).

17. W. Liang, V. Ilchenko, D. Eliyahu, A. Savchenkov, A. Matsko, D. Seidel, L. Maleki, Ultralow noise miniature external cavity semiconductor laser. *Nat. Commun.* **6**, 7371 (2015).

18. A. Savchenkov, W. Zhang, V. Iltchenko, A. Matsko, Robust self-injection locking to a non-confocal monolithic Fabry–Perot cavity. *Opt. Lett.* **49**, 1520–1523 (2024).

19. S. V. Pericherla, L. Trask, C. Shirpurkar, A. Bhardwaj, G. E. Hoefler, P. J. Delfyett, Stabilization of an InP mode-locked laser PIC through simultaneous optical filtering and self-injection locking using a fabry-perot etalon. *J. Lightwave Technol.* **42**, 3573–3579 (2024).

20. V. V. Spirin, J. L. Bueno Escobedo, D. A. Korobko, P. Mégret, A. A. Fotiadi, Stabilizing DFB laser injection-locked to an external fiber-optic ring resonator. *Opt. Express* **28**, 478–484 (2020).

21. T. Aihara, T. Hiraki, T. Fujii, K. Takeda, T. Tsuchizawa, T. Kakitsuka, H. Fukuda, S. Matsuo, Heterogeneously integrated widely tunable laser using lattice filter and ring resonator on Si photonics platform. *Opt. Express* **30**, 15820–15829 (2022).

22. H. Guan, A. Novack, T. Galfsky, Y. Ma, S. Fathololoumi, A. Horth, T. N. Huynh, J. Roman, R. Shi, M. Caverley, Y. Liu, T. Baehr-Jones, K. Bergman, M. Hochberg, Widely-tunable, narrow-linewidth III-V/silicon hybrid external-cavity laser for coherent communication. *Opt. Express* **26**, 7920–7933 (2018).

23. C. Li, S. Sui, F. Gao, Y. Wang, X. Xu, J. Zhao, High-power III-V/Si integrated wavelength tunable laser for L-band applications. *IEEE J. Quantum Electron.* **59**, 2000606 (2023).

24. Z. Li, R. N. Wang, G. Lihachev, J. Zhang, Z. Tan, M. Churaev, N. Kuznetsov, A. Siddharth, M. J. Bereyhi, J. Riemensberger, T. J. Kippenberg, High density lithium niobate photonic integrated circuits. *Nat. Commun.* **14**, 4856 (2023).

25. M. Li, L. Chang, L. Wu, J. Staffa, J. Ling, U. A. Javid, S. Xue, Y. He, R. Lopez-rios, T. J. Morin, H. Wang, B. Shen, S. Zeng, L. Zhu, K. J. Vahala, J. E. Bowers, Q. Lin, Integrated Pockels laser. *Nat. Commun.* **13**, 5344 (2022).

26. M. Zhang, C. Wang, R. Cheng, A. Shams-Ansari, M. Lončar, Monolithic ultra-high-Q lithium niobate microring resonator. *Optica* **4**, 1536–1537 (2017).

27. G. Lihachev, J. Riemensberger, W. Weng, J. Liu, H. Tian, A. Siddharth, V. Snigirev, V. Shadymov, A. Voloshin, R. N. Wang, J. He, S. A. Bhave, T. J. Kippenberg, Low-noise frequency-agile photonic integrated lasers for coherent ranging. *Nat. Commun.* **13**, 3522 (2022).

28. Q. Su, F. Wei, C. Chen, Z. Fang, H. Pi, Y. Sun, F. Yang, A. Stroganov, H. Wu, G. Xin, X. Chen, F. Yang, Q. Ye, H. Cai, W. Chen, A self-injection locked laser based on high-Q micro-ring resonator with adjustable feedback. *J. Lightwave Technol.* **41**, 6756–6763 (2023).

29. A. Prokoshin, M. Gehl, S. Madaras, W. W. Chow, Y. Wan, Ultra-narrow-linewidth hybrid-integrated self-injection locked laser at 780 nm. *Optica* **11**, 1024–1029 (2024).

30. A. S. Voloshin, N. M. Kondratiev, G. V. Lihachev, J. Liu, V. E. Lobanov, N. Y. Dmitriev, W. Weng, T. J. Kippenberg, I. A. Bilenko, Dynamics of soliton self-injection locking in optical microresonators. *Nat. Commun.* **12**, 235 (2021).

31. T. Wildi, A. E. Ulanov, T. Voumard, B. Ruhnke, T. Herr, Phase-stabilised self-injection-locked microcomb. *Nat. Commun.* **15**, 7030 (2024).

32. Y. Guo, R. Zhao, G. Zhou, L. Lu, A. Stroganov, M. S. Nisar, J. Chen, L. Zhou, Thermally tuned high-performance III-V/$Si_3N_4$ external cavity laser. *IEEE Photonics J.* **13**, 1–13 (2021).

33. A. Blanco-Redondo, I. Andonegui, M. J. Collins, G. Harari, Y. Lumer, M. C. Rechtsman, B. J. Eggleton, M. Segev, Topological optical waveguiding in silicon and the transition between topological and trivial defect states. *Phys. Rev. Lett.* **116**, 163901 (2016).

34. S. Weimann, M. Kremer, Y. Plotnik, Y. Lumer, S. Nolte, K. G. Makris, M. Segev, M. C. Rechtsman, A. Szameit, Topologically protected bound states in photonic parity–time-symmetric crystals. *Nat. Mater.* **16**, 433–438 (2017).

35. Z.-K. Shao, H.-Z. Chen, S. Wang, X.-R. Mao, Z.-Q. Yang, S.-L. Wang, X.-X. Wang, X. Hu, R.-M. Ma, A high-performance topological bulk laser based on band-inversion-induced reflection. *Nat. Nanotechnol.* **15**, 67–72 (2020).

36. X. Sun, Z. Li, Y. Sun, Y. Wang, J. Wang, J. H. Marsh, S. J. Sweeney, A. E. Kelly, L. Hou, Narrow linewidth laser based on extended topological interface states in 1D photonic crystals. *Laser Photonics Rev.* **19**, 2500383 (2025).

37. L. Hou, P. Stolarz, J. Javaloyes, R. P. Green, C. N. Ironside, M. Sorel, A. C. Bryce, Subpicosecond pulse generation at quasi-40-GHz using a passively mode-locked AlGaInAs–InP 1.55-μm strained quantum-well laser. *IEEE Photonics Technol. Lett.* **21**, 1731–1733 (2009).

38. L. B. Mercer, 1/f Frequency noise effects on self-heterodyne linewidth measurements. *J. Lightwave Technol.* **9**, 485–493 (1991).

39. M. A. Tran, D. Huang, J. E. Bowers, Tutorial on narrow linewidth tunable semiconductor lasers using Si/III-V heterogeneous integration. *APL Photonics* **4**, 111101 (2019).

40. Z. Zhao, Z. Bai, D. Jin, Y. Qi, J. Ding, B. Yan, Y. Wang, Z. Lu, R. P. Mildren, Narrow laser-linewidth measurement using short delay self-heterodyne interferometry. *Opt. Express* **30**, 30600–30610 (2022).

41. G. L. Roberts, M. Lucamarini, J. F. Dynes, S. J. Savory, Z. Yuan, A. J. Shields, Modulator-free coherent-one-way quantum key distribution. *Laser Photonics Rev.* **11**, 1700067 (2017).

42. T. K. Paraïso, I. De Marco, T. Roger, D. G. Marangon, J. F. Dynes, M. Lucamarini, Z. Yuan, A. J. Shields, A modulator-free quantum key distribution transmitter chip. *NPJ Quantum Inf.* **5**, 42 (2019).

43. D. A. Heim, D. Bose, K. Liu, A. Isichenko, D. J. Blumenthal, Hybrid integrated ultra-low linewidth coil stabilized isolator-free widely tunable external cavity laser. *Nat. Commun.* **16**, 5944 (2025).

44. H. Cheng, C. Xiang, N. Jin, I. Kudelin, J. Guo, M. Heyrich, Y. Liu, J. Peters, Q.-X. Ji, Y. Zhou, K. J. Vahala, F. Quinlan, S. A. Diddams, J. E. Bowers, P. T. Rakich, Harnessing micro-Fabry–Pérot reference cavities in photonic integrated circuits. *Nat. Photonics* **8**, eabp9006 (2025).

45. N. M. Kondratiev, M. L. Gorodetsky, Thermorefractive noise in whispering gallery mode microresonators: Analytical results and numerical simulation. *Phys. Lett. A* **382**, 2265–2268 (2018).

46. G. Zhang, Q. Cen, T. Hao, X. Yin, X. Zi, N. Shi, W. Li, N. Zhu, M. Li, Self-injection locked silica external cavity narrow linewidth laser. *J. Lightwave Technol.* **41**, 2474–2483 (2023).

47. Y. Xu, P. Maier, M. Blaicher, P.-I. Dietrich, P. Marin-Palomo, W. Hartmann, Y. Bao, H. Peng, M. R. Billah, S. Singer, U. Troppenz, M. Moehrle, S. Randel, W. Freude, C. Koos, Hybrid external-cavity lasers (ECL) using photonic wire bonds as coupling elements. *Sci. Rep.* **11**, 16426 (2021).

48. J. Wang, X. Li, X. Guo, T.-H. Loh, L. Ranno, C. Liu, Rusli, H. Wang, J. X. Brian Sia, Scalable single-microring hybrid III-V/Si lasers for emerging narrow-linewidth applications. *Opt. Express* **32**, 26751–26762 (2024).

49. Y. Liu, Y. Chen, L. Bogaert, E. Soltanian, E. Delli, G. Lepage, P. Verheyen, J. Van Campenhout, G. Morthier, G. Roelkens, J. Zhang, Widely tunable narrow-linewidth lasers with booster amplification on silicon photonics. *Opt. Express* **33**, 22078–22086 (2025).

50. C. Xiang, W. Jin, J. Guo, J. D. Peters, M. Kennedy, J. Selvidge, P. A. Morton, J. E. Bowers, Narrow-linewidth III-V/Si/$Si_3$N4 laser using multilayer heterogeneous integration. *Optica* **7**, 20–21 (2020).

51. K. A. McKinzie, C. Wang, A. A. Noman, D. L. Mathine, K. Han, D. E. Leaird, G. E. Hoefler, V. Lal, F. Kish, M. Qi, A. M. Weiner, InP high power monolithically integrated widely tunable laser and SOA array for hybrid integration. *Opt. Express* **29**, 3490–3502 (2021).

52. Y. Zhu, S. Yu, Z. Fang, D. Yin, J. Liu, Z. Wang, Y. Zhou, Y. Ma, H. Zhang, M. Wang, Y. Cheng, Integrated electro-optically tunable narrow-linewidth III–V laser. *Adv. Photon Res.* **5**, 2400018 (2024).

53. M. Han, J. Li, H. Yu, D. Li, R. Li, J. Liu, Integrated self-injection-locked narrow linewidth laser based on thin-film lithium niobate. *Opt. Express* **32**, 5632–5640 (2024).

54. A. Siddharth, S. Bianconi, R. N. Wang, Z. Qiu, A. S. Voloshin, M. J. Bereyhi, J. Riemensberger, T. J. Kippenberg, Ultrafast tunable photonic-integrated extended-DBR Pockels laser. *Nat. Photonics* **19**, 709–717 (2025).

55. M. C. Larson, A. Bhardwaj, W. Xiong, Y. Feng, X. Huang, K. Petrov, M. Moewe, H. Ji, A. Semakov, C. Lv, S. Kutty, A. Patwardhan, N. Liu, Z. M. Li, Y. J. Bao, Z. H. Shen, S. Bajwa, F. H. Zhou, P. C. Koh, "Narrow Linewidth sampled-grating distributed Bragg reflector laser with enhanced side-mode suppression," in *Optical Fiber Communication Conference*, OSA Technical Digest (online) (Optica Publishing Group, 2015), pp. M2D. 1.

56. Y. Xu, M. Wang, H. Qu, W. Liu, T. Fu, J. Li, F. Du, W. Zheng, Narrow-linewidth semiconductor laser with high-order sidewall gratings. *IEEE Photonics Technol. Lett.* **35**, 85–88 (2022).

57. R. R. Kumar, A. Hänsel, M. Far Brusatori, L. Nielsen, L. M. Augustin, N. Volet, M. J. Heck, A 10-kHz intrinsic linewidth coupled extended-cavity DBR laser monolithically integrated on an InP platform. *Opt. Lett.* **47**, 2346–2349 (2022).

58. E. D. Gaetano, M. Sorel, Design of chirped-coupling sidewall Bragg gratings for narrow linewidth distributed feedback lasers. *Opt. Lett.* **44**, 1642–1645 (2019).

59. M. Hammer, K. R. Hiremath, R. Stoffer, "Analytical approaches to the description of optical microresonator devices," in *AIP Conference Proceedings* (American Institute of Physics, 2004), vol. 709, pp. 48–71.

60. M. Chin, S. Ho, Design and modeling of waveguide-coupled single-mode microring resonators. *J. Lightwave Technol.* **16**, 1433–1446 (1998).

61. G. Mezosi, *Semiconductor Ring Lasers for All-Optical Signal Processing* (University of Glasgow, 2011).

62. F. D. M. Haldane, S. Raghu, Possible realization of directional optical waveguides in photonic crystals with broken time-reversal symmetry. *Phys. Rev. Lett.* **100**, 013904 (2008).

63. M. Xiao, Z. Zhang, C. T. Chan, Surface impedance and bulk band geometric phases in one-dimensional systems. *Phys. Rev. X* **4**, 021017 (2014).

64. N. Kondratiev, V. Lobanov, A. Cherenkov, A. Voloshin, N. Pavlov, S. Koptyaev, M. Gorodetsky, Self-injection locking of a laser diode to a high-Q WGM microresonator. *Opt. Express* **25**, 28167–28178 (2017).
